# Safety and efficacy assessment of standardized herbal formula PM012

**DOI:** 10.1186/1472-6882-12-24

**Published:** 2012-03-29

**Authors:** Sung-Hwa Sohn, Soo-Jeong Kim, Yong Kim, Insop Shim, Hyunsu Bae

**Affiliations:** 1 Department of Physiology, College of Oriental Medicine, Kyung Hee University, Hoegi-dong, Seoul, Dongdaemun-gu 130-701, Republic of Korea; 2 Present address: Department of Psychiatry, Seoul St. Mary's Hospital, College of Medicine, The Catholic University of Korea, 505 Banpo-dong, Seoul, Seocho-gu 137-701, Republic of Korea

**Keywords:** PM012, Repeated oral toxicity, Presenilin 2, NOAEL, Morris water maze, Spatial memory

## Abstract

**Background:**

This study was conducted to evaluate the efficacy of the herbal formula PM012 on an Alzheimer's disease model, human presenilin 2 mutant transgenic mice (hPS2m), and also to evaluate the toxicity of PM012 in Sprague-Dawely rats after 4 or 26 weeks treatment with repeated oral administration.

**Methods:**

Spatial learning and memory capacities of hPS2m transgenic mice were evaluated using the Morris Water Maze. Simultaneously, PM012 was repeatedly administered orally to male and female SD rats (15/sex/group) at doses of 0 (vehicle control), 500, 1,000 and 2,000 mg/kg/day for 4 or 26 weeks. To evaluate the recovery potential, 5 animals of each sex were assigned to vehicle control and 2,000 mg/kg/day groups during the 4-week recovery period.

**Results:**

The results showed that PM012-treated hPS2m transgenic mice showed significantly reduced escape latency when compared with the hPS2m transgenic mice. The repeated oral administration of PM012 over 26 weeks in male and female rats induced an increase and increasing trend in thymus weight in the female treatment groups (main and recovery groups), but the change was judged to be toxicologically insignificant. In addition, the oral administration of the herbal medicine PM012 did not cause adverse effects as assessed by clinical signs, mortality, body weight, food and water consumption, ophthalmology, urinalysis, hematology, serum biochemistry, blood clotting time, organ weights and histopathology. The No Observed Adverse Effects Levels of PM012 was determined to be 2,000 mg/kg/day for both sexes, and the target organ was not identified.

**Conclusion:**

These results suggest that PM012 has potential for use in the treatment of the Alzheimer's disease without serious adverse effects.

## Background

Alzheimer's disease (AD) is the major cause of dementia and underlies more than 60% of dementia cases [1]. AD occurs more frequently in older age groups [2]. AD is characterized by the impairment of cognitive performance such as attention, memory and learning, in addition to changes in cholinergic markers, including levels of acetylcholine (ACh) and choline acetyltransferase (ChAT) [3-5]. In addition, β-amyloid precursor protein (*β-APP*), Presenilin 1 (PS1), Presenilin 2 (PS2), and apolipoprotein E type 4 (*APOE-E4*) mutations are linked to the early onset of AD [6-10]. Of these, the mutated PS genes were observed in the highest proportion of early AD patients [6,11].

Herbal medicines are used for the prevention and treatment of various diseases. For example, *Ginkgo biloba, Ginseng *and *Melisa officinalis *have been commonly used as memory or cognition enhancers. The effects of these enhancers have been demonstrated scientifically [12-14]. Yukmijihwang-tang or Luweidihuang-wang (YMJ) is composed of 6 herbal medicines, *Corni fructus, Rehmannia radix, Hoelen, Discoreae radix, Mountain cortex radicis *and *Alismatis radix*. YMJ has long been applied to the treatment of diabetes mellitus and neurosis. Ancient Chinese herbal textbooks also reference YMJ as an anti-aging treatment. In a previous study, YMJ derivatives (PM012) containing *Lycii fructus*, in addition to the other components of YMJ, enhanced memory retention by protecting neuronal cells and enhancing cell proliferation and neurite growth [15]. In addition, PM012 accelerated the speed of information processing and enhanced cognitive abilities in normal subjects [16]. It has also been reported that PM012 treatment prevents the loss of cholinergic cells through anti-oxidative and anti-inflammatory effects, recovers reduced ChAT in the medial septum and improves overall spatial learning ability and the working memory deficits produced by ibotenic acid [17-19]. However, because the chemically induced dementia models do not mimic the pathophysiology of Alzheimer's disease in humans, it is necessary to evaluate a more clinically relevant and straightforward disease model such as hPS2m transgenic mice. Further, there are no reports regarding the safety of YMJ or PM012 to date. Therefore, we here report the results of our investigation of the safety and efficacy of the PM012 extract in rodents. This study was conducted in compliance with the Good Laboratory Practice (GLP) [20] and Test Guidelines of the Organization for Economic Cooperation and Development (OECD) [21] and U.S. Food and Drug Administration (FDA)[22] and the Korea Food and Drug Administration (KFDA) [23].

## Methods

### Transgenic mice

The human presenilin 2 (hPS2) mutant (N141I) transgenic mice (hPS2m) were generously provided by the National Institute of Toxicological Research (Korea FDA) and housed in a controlled environment (12:12-hour light:dark cycle, temperature 23 ± 2°C, humidity 50 ± 10%) with water and food available *ad libitum*. The mice were randomly assigned to two groups (4-5 mice in each group), defined as follows: group 1: hPS2m tg; group 2: hPS2m tg + drug treatment. The hPS2m transgenic mice received a commercial mice chow, while the drug treated-hPS2m transgenic mice had access to chow containing 2% PM012. They were each fed their respective chow from 32 to 48 weeks of age during the growing period. All experiments in this study were approved by the Institutional Animal Care and Use Committee of Kyung Hee University.

### Morris water maze test

A modified version of the procedure described by Morris was used [24]. The water maze was a circular pool 1.5 m in diameter, constructed of fiberglass. The pool contained water maintained at a temperature of 22 ± 2°C and contained 1 kg of powdered skim milk to make the water opaque. During the testing in the water maze, a platform (15 cm in diameter) was fixed at 1 cm below the surface of the water at identical locations within the pool. The pool was surrounded by different extra-maze cues. Each trial was initiated at one of different starting positions and the route taken out of the pool and swimming path of each rat were recorded with a video camera connected to a video recorder and a tracking device (S-MART, Pan-Lab, Spain). All mice were subjected to four trials per day at intervals of 15 min for four consecutive days followed by one day of probe trials on the fifth day. The trials were considered to be completed when the rat found the hidden platform or the escape latency reached 60 s. For the probe trial, the platform was removed from the pool and the rat was allowed to swim freely for 60 s to search for the previous location of the platform. The proportion of time spent searching for the platform in the training quadrant, i.e. the previous location of the platform, was used as a measure of memory retention.

### Animal maintenance for the toxicity test

According to the Korea FDA guideline, we selected Sprague Dawley (SD) rats for toxicity assessment. Seventy male and seventy female SD rats, 5 weeks old, were obtained from Koatec Co. Ltd (Gyeonggi-do, Korea). Rats were acclimated in a controlled room (temperature: 23 ± 3°C, relative humidity: 55 ± 15%, air circulating frequency: 10-20 times/hr, artificial light: from 8 am to 8 pm) for 7 days before experimentation. Rats were housed in stainless steel cages (W215 × L355 × H 200 mm). Animals were offered irradiation-sterilized pellet food for lab animals (Harlan Co. Ltd, USA), purchased from FOLAS international Inc., *ad libitum*. According to the certificates evaluating the diet components and contaminants supplied by the diet provider, there were no factors in the chow that could affect the results of this study. Groundwater disinfected by ultraviolet sterilizer and ultrafiltration was given via water bottle *ad libitum*. The experimental protocols for this study were approved by the Institutional Animal Care and Use Committee, and the animals were cared for in accordance with the institutional ethical guidelines.

### Preparation of PM012

Water-extracted dried herbal medicines were purchased from Sun Ten Pharmaceutical (Taipei, Taiwan). The ratio of each component in PM012 is shown in Table 1. The amount of standard phytochemicals of each herbal medicine was determined by an HPLC-based quantification method (Table 1).

**Table 1:** The contents of PM012 and the amounts of standard materials

Herbal medicines	Ratio (%)	Standard materials	Standard material contents (mg/PM012 g ext.)
*Lycii fructus*	4 (26.5%)	Betain	0.32 ± 0.02 (0.03%)
*Rehmannia radix*	4 (26.5%)	5-HMF	0.20 ± 0.02 (0.02%)
*Corni fructus*	2 (13%)	Loganin	1.29 ± 0.05 (0.13%)
*Discoreae radix*	2 (13%)	Allantoin	1.31 ± 0.21 (0.13%)
*Hoelen*	1 (7%)		
*Alismatis radix*	1 (7%)		
*Mountain cortex radicis*	1 (7%)	Paeonol	0.93 ± 0.033 (0.09%)

### Experimental design for toxicity test

Animals were randomly divided into six groups consisting of 15 or 5 rats of each gender. Group I animals (control) were administered distilled water (DW) by gavage throughout the course of the study. Animals in Groups II (500 mg/kg body weight/day), III (1000 mg/kg body weight/day), IV (2000 mg/kg body weight/day), V (recovery control) and VI (recovery group, 2000 mg/kg body weight/day) received orally administered PM012 dissolved in DW by gastric intubation for a period of 4 or 26 weeks. Urinalyses were conducted during the last 4 days of the administration period. The urine of each group of animals was collected for 24 h and the volume of urine was measured. Animals were individually placed in metabolic cages in batches for a period of 24 h and were provided with water but not food. The animals were only fasted in metabolic cages for a period of 24 h. Food and water were provided *ad libitum *during the other 3 days of sampling of the other groups. Following the observation period, all animals were anesthetized with 5 mg/kg of ketamine HCl intramuscularly (Ketamine^®^, Yuhan Co., Korea). An autopsy was conducted on every animal and all major organs and tissues including heart, lung, liver, stomach, intestine, kidney, adrenal gland, spleen and ovary or testicle were examined for gross lesions.

### Clinical observations and survival

Rats were observed twice daily (morning and afternoon) for signs of clinical toxicity and mortality. Body weights were recorded daily throughout the study period. Mean daily food consumption was calculated each day by subtracting the weight of the remaining food from the weight of the supplied food. Clinical examinations were performed twice daily, first at the time of dose administration and again approximately 1-2 h following dose administration. Observations included changes in skin, fur, eyes, oral mucosa, respiration, circulation and behavior.

### Urinalysis

In the last week of observation, 15 animals per group were housed in a metabolic cage for urine collection, and approximately 1 ml of fresh urine collected over 3-4 hours was analyzed by urinalysis test strips (Multistix 10SG, Bayer, USA) and an automatic analyzing instrument (CliniTek 100, Bayer, USA). The following parameters were analyzed: glucose, bilirubin, ketone bodies, specific gravity, occult blood, pH, protein, urobilinogen, nitrite and leukocytes. Samples were also examined microscopically for the presence of urinary sediments, including erythrocytes, leukocytes, epithelial cells and cast.

### Hematology and serum biochemistry

Blood samples were measured for clotting time, red blood cell (RBC) and white blood cell (WBC) counts, hemoglobin (Hb), hematocrit (HCT), mean corpuscular volume (MCV), mean corpuscular Hb (MCH), mean corpuscular Hb concentration (MCHC), red cell distribution width (RDW), Hb concentration distribution width (HDW), reticulocytes (RET), platelets (PLT), mean platelet volume (MPV), large unstained cells (LUC), neutrophils (NEU), lymphocytes (LYM), monocytes (MONO), eosinophils (EOS), and basophils (BASO) with a Coulter counter (ADVIA 2120, SIEMENS, USA) according to the manufacturer's operator manual. Serum from blood samples collected in separator tubes was analyzed for changes in biochemistry using an AU400 Serum biochemistry analyzer (AU400, Olympus, Japan), which measured aspartate aminotransferase activity (AST), alanine aminotransferase activity (ALT), alkaline phosphatase activity (ALP), creatine phosphokinase activity (CPK), total bilirubin (BIL), glucose (GLU), total cholesterol (CHO), triglycerides (TG), total protein (PRO), albumin (ALB), the albumin/globulin ratio (A/G ratio), blood urea nitrogen (BUN), creatinine (CRE), and inorganic phosphorus (IP). The concentrations of calcium ions (Ca^2+^), sodium ions (Na^+^), potassium ions (K^+^), and chloride ions (Cl^-^) were measured with an electrolyte autoanalyzer (RAPIDCHEM 744 Na^+^/K^+^/Cl^- ^Analyzer, SIEMENS, USA).

### Blood clotting time assessment

A total of 1.8 mL of blood was dispensed into a microtube containing 0.2 mL 3.2% sodium citrate. Plasma was obtained by centrifugation at 735 × g (5402, Eppendorf, Germany, 3,000 rpm, 735 RCF) at 4°C for 10 min. Prothrombin time (PT) and activated partial thromboplastin time (APTT) were measured in seconds from the plasma using the nephelometric analysis method with a coagulation time analyzer (ACL 100, Instrumentation Laboratory, USA).

### Organ weights

During the necropsy, the organs such as ovaries (both), uterus, adrenal glands (both), pituitary gland, thymus, prostate gland, testes (both), epididymides (both), spleen, kidneys (both), heart, lungs, brain, and liver were weighed with an electronic balance, and all the paired organs were measured separately. The weights of these organs were converted to relative organ weights based on the organ-to-body weight ratio.

### Fixation and storage of organs

Eye balls were fixed in Davidson's solution, and testes and epididymides were fixed in Bouin's solution. The following organs of all animals were fixed in 10% neutral formalin: testes, epididymides, seminal vesicles, prostate gland, ovaries, uterus, vagina, urinary bladder, spleen, stomach, pancreas, duodenum, jejunum, ileum, cecum, colon, rectum, mesenteric lymph node, adrenal glands, kidneys, liver, skeletal muscle, sciatic nerve, femur, submandibular lymph nodes, salivary glands, sternum, thymus, heart, lungs, aorta, thoracic spinal cord, tongue, trachea, esophagus, thyroid glands, harderian glands, eyes, brain, pituitary gland, and skin (mammary glands).

### Histopathological assessment

The fixed organs of the vehicle control and high dose group and any organs from the other groups that displayed gross abnormalities were subjected to histopathological examination. Tissues were embedded in paraffin and microsections of 4-5 μm were taken from the block. Hematoxylin & Eosin-stained slides were prepared and the specimens were microscopically examined with an optical microscope.

### Statistical analysis

Statistical analysis of the data was conducted using SPSS 10.1. Data were analyzed by an unpaired *t*-test or two-way ANOVA or one-way analysis of variance (ANOVA) followed by the Dunnett multiple comparisons test. Results with a p-value < 0.05 were considered statistically significant.

## Results

### Efficacy assessment

#### Effects of PM012 on the body weight of hPS2m transgenic mice

**Figure 1:**
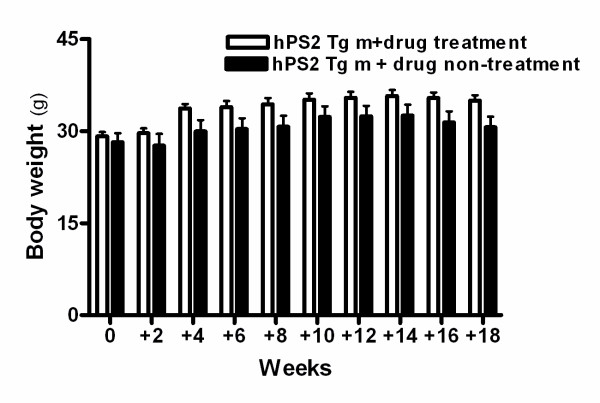
**Change of the body weight of the experimental mice**. The body weight of the hPS2 transgenic mice and the drug treated-hPS2 transgenic mice at 0, 2, 4, 6, 8, 10, 12, 14, 16 and 18 days following drug treatment (n = 4-5/group). The data shown are the means ± S.E.M (n = 7-8/group).

To determine whether PM012 treatment causes side effects, we measured its effects on body weight. As shown in Figure 1, there were significant differences in body weight between the hPS2m transgenic mice and the drug treated-hPS2m transgenic mice [F (1, 70) = 21.21, p < 0.001]. The increase in body weight over the experimental period was significantly higher in the drug treated-hPS2 transgenic mice compared to the hPS2m transgenic mice (*t*-test; t = 3.387, df = 18, p = 0.0033). The average body weight of the hPS2m transgenic mice versus the drug treated-hPS2m transgenic mice was 23.180 ± 1.476 g versus 29.175 ± 0.638 g at 0 week, 27.620 ± 1.930 g versus 29.625 ± 0.819 g at 2 weeks, 29.940 ± 1.857 g versus 33.675 ± 0.735 g at 4 weeks, 30.320 ± 1.777 g versus 33.900 ± 0.998 g at 6 weeks, 30.680 ± 1.810 g versus 34.375 ± 1.005 g at 8 weeks, 32.300 ± 1.731 g versus 35.100 ± 1.027 g at 10 weeks, 32.420 ± 1.704 g versus 35.400 ± 1.002 g at 12 weeks, 32.540 ± 1.745 g versus 35.700 ± 0.99 g at 14 weeks, 31.420 ± 1.774 g versus 35.375 ± 0.923 g at 16 weeks and 31.420 ± 1.774 g versus 34.975 ± 0.863 g at 18 weeks.

#### PM012 reverses spatial learning deficits at 12 months of age in hPS2m transgenic mice

**Figure 2:**
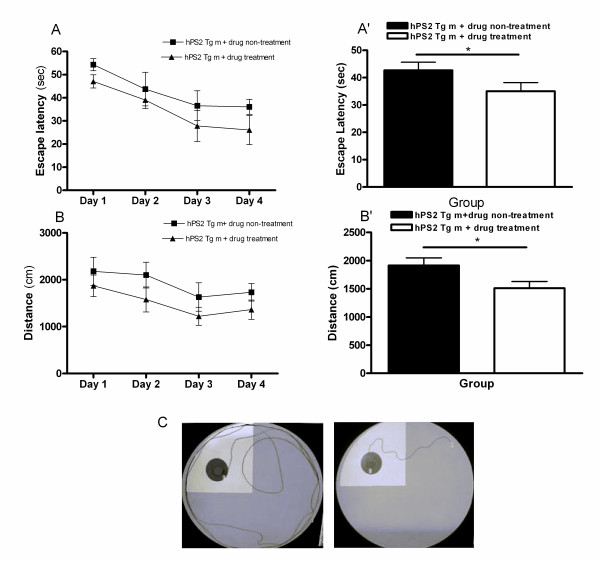
**Alteration of escape latency and swimming distance during an acquisition test using the water maze**. Four trials per day for 4 consecutive days were conducted and the escape latency (**A and A'**) and swimming distance (**B and B'**) were measured. Representative images (C) showing the swimming path of mice during the tests. Left: drug non treated- hPS2 transgenic mice and right: drug treated-hPS2 transgenic mice. Data are presented as mean ± SEM, and comparisons between the two groups were evaluated by the unpaired *t*-test. *P < 0.05 compared to the drug treated-hPS2 transgenic mice.

To determine whether PM012 treatment affects spatial memory tasks, we evaluated its effects on learning and memory in hPS2m transgenic mice using the Morris water maze test. In this study, drug treatment had a significant effect on escape latency (i.e., the swimming time required to find a hidden platform in the acquisition test) and total swimming distance (i.e., the swim path length taken to find the hidden platform in the acquisition test) among the hPS2m transgenic mice. The memory deficiency exhibited by mice with the hPS2m mutation was significantly alleviated in the drug treated-hPS2m transgenic mice when compared with the untreated hPS2m transgenic mice. As shown in Figure 2A and 2A', analysis of the escape latency revealed a significant difference between groups [F (1, 21) = 2.963, p < 0.05] and depending on the time of day [F (3, 21) = 5.9, p < 0.01]. On days 1-4, the drug treated-hPS2 transgenic mice showed a significantly reduced escape latency when compared with the hPS2 transgenic mice (*t*-test; t = 1.762, df = 34, p = 0.0438). As shown in Figure 2B and 2B', the total swimming distance differed significantly among groups [F (1, 21) = 7.70, p < 0.05]. On days 1-4, the drug treated-hPS2 transgenic mice showed a significantly reduced escape latency when compared with the hPS2 transgenic mice (*t*-test; t = 2.183, df = 34, p = 0.0180). Conversely, there was no significant difference in the average swimming speed among groups on all training days (data not shown).

**Figure 3:**
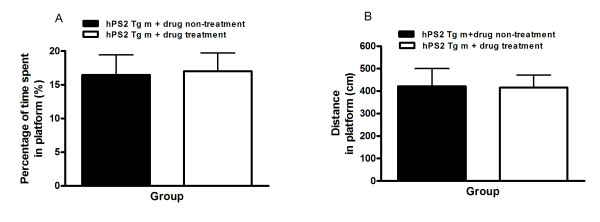
**Alteration of time spent and distance around the platform and during the memory retention test in the water maze**. Four trials were conducted on the fifth day without the platform and the search latency (**A**) and distance (**B**) were measured. Data are presented as mean ± SEM, and comparisons between the two groups were made using the unpaired *t*-test. *P < 0.05, compared to the drug treated-hPS2 transgenic mice.

The performance on the probe trial, which compared the percentage of time spent swimming around the platform on day 5, is illustrated in Figure 3A. There was no significant difference in time spent swimming around the platform among the mice (Figure 3B). The learning and memory retention performance test revealed that the drug treated-hPS2 transgenic mice spent a slightly longer time around the platform than the hPS2 transgenic mice, but this difference was not significant (*t*-test; t = 0.1253, df = 7, p = 0.452). These findings indicated that PM012 treatment ameliorated spatial memory impairment in hPS2m mice without affecting locomotive activity.

### Safety assessment

#### Clinical observations and survival

**Figure 4:**
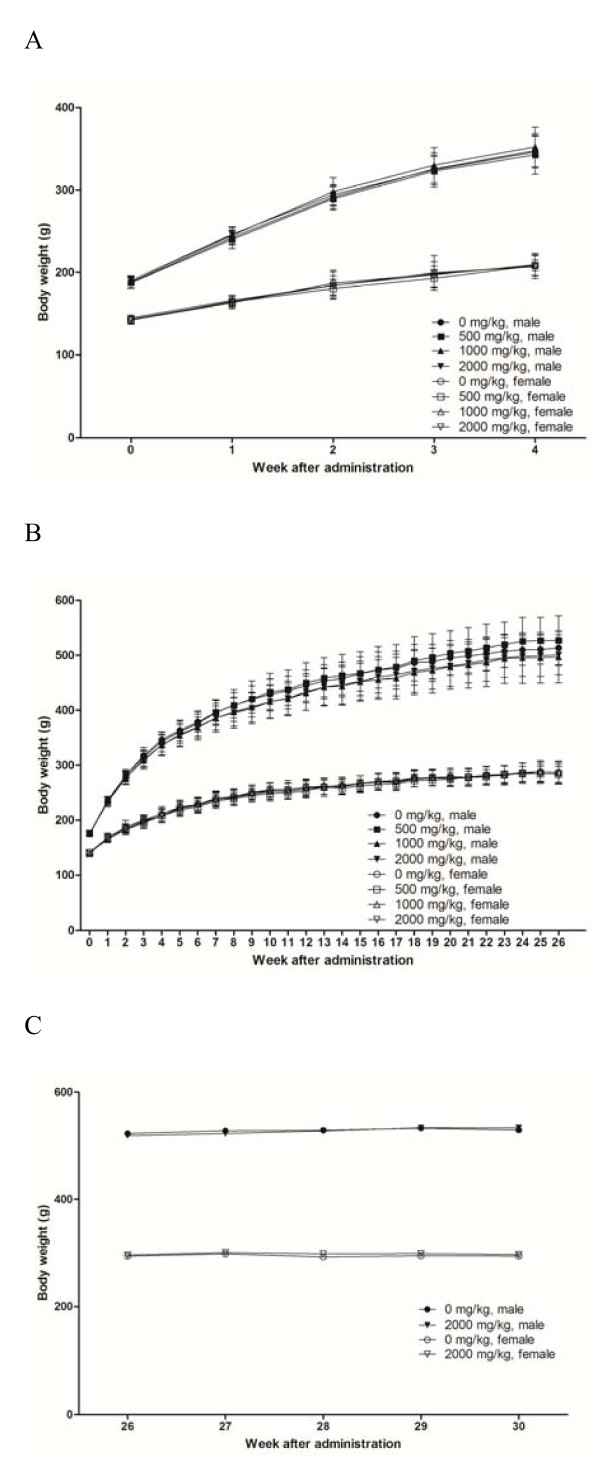
**Mean body weight of male and female rats dosed with PM012**. Each data point represents the mean body weight of groups of ten or fifteen animals. A) main group receiving 4 weeks of treatment, B) main group receiving 26 weeks of treatment and C) 4 weeks recovery group. Statistical analysis of the data (Dunnett's test) revealed no significance differences between groups.

**Figure 5:**
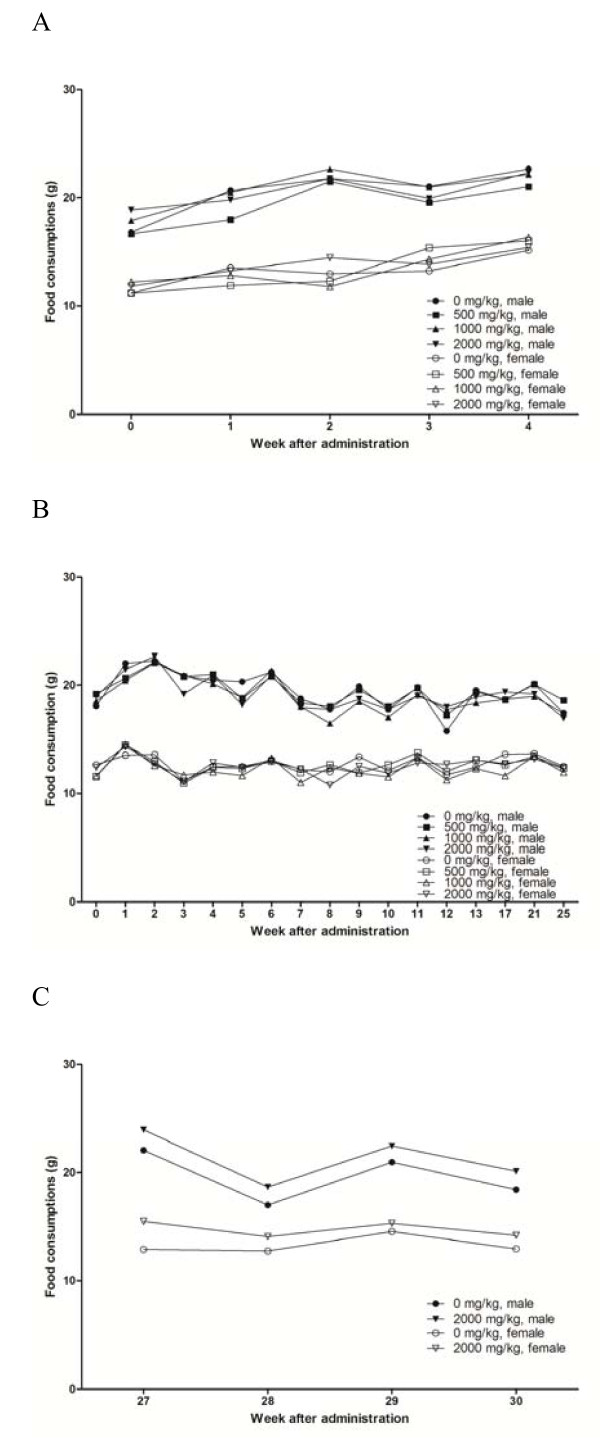
**Mean food consumption by male and female rats treated with PM012**. Each data point represents the mean food consumption of groups of ten or fifteen animals. A) main group receiving 4 weeks of treatment, B) main group receiving 26 weeks of treatment and C) 4 weeks recovery group. Statistical analysis of the data (Dunnett's test) revealed no significance differences between groups.

**Figure 6:**
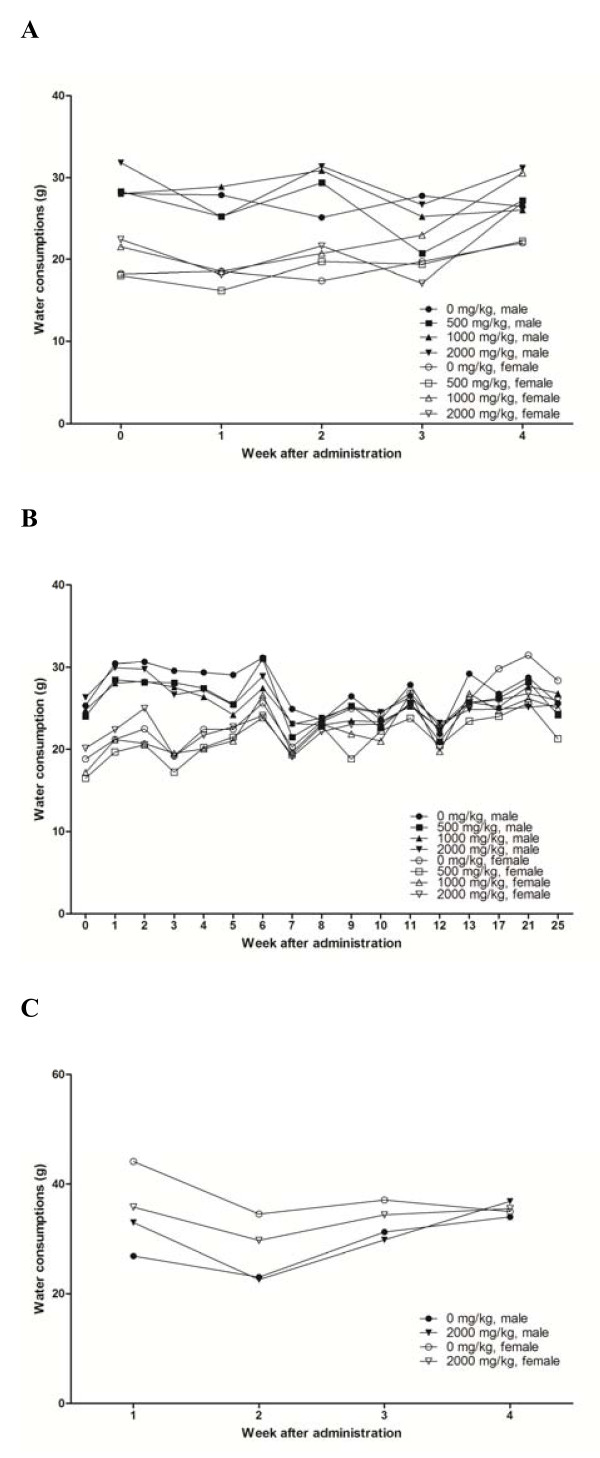
**Mean water consumption by male and female rats treated with PM012**. Each data point represents the mean water consumption of groups of ten or fifteen animals. A) main group receiving 4 weeks of treatment, B) main group receiving 26 weeks of treatment and C) 4 weeks recovery group. Statistical analysis of the data (Dunnett's test) revealed no significance differences between groups.

The rats from all treatment groups appeared to be healthy at the conclusion of the study period (Table 2). In general, there were no statistically significant changes in body weights (Figure 4). There were no significant differences in food (Figure 5) and water (Figure 6) consumption between the control and treatment groups. In addition, there were no statistically significant differences in food and water consumption, regardless of sex or recovery group (Figure 5 and 6). No abnormal findings were found in the ophthalmic examination (data not shown).

**Table 2:** Abnormal clinical signs and mortality in rats orally treated with PM012

Sex	Dose (mg/kg)	Observations	
		
		0-4 Weeks	0-26 Weeks
Male	0	Appears normal	Appears normal
	500	Appears normal	Appears normal
	1000	Appears normal	Appears normal
	2000	Appears normal	Appears normal
Female	0	Appears normal	Appears normal
	500	Appears normal	Appears normal
	1000	Appears normal	Appears normal
	2000	Appears normal	Appears normal

#### Urinalysis

Semi-quantitative urinary examinations, such as pH, specific gravity, protein, glucose, urea acid, ketone and occult blood, did not reveal any relevant changes following acute administration of the PM012 extract (Additional file 1: Table S1). Moreover, PM012 treatment did not cause any significant changes in the presence of urinary sediments (data not shown). In recovery groups, the urine volume in males treated with 2,000 mg/kg/day was significantly (P < 0.05) higher than that in the vehicle control group.

#### Hematology

Hematology data are summarized in Table 3. No statistically significant differences were observed in the hematology parameters, including RBC, PLT, WBC, NEU, LYM, MONO, EOS, BASO, and LUC counts upon PM012 treatment. Similarly, there were no significant changes in clotting time, HDW, Hb, RET, HCT, HGB, MCV, RDW, MCH, MCHC and MPV values between the control and treated animals (main groups). In recovery groups, the increases in MCV, MCH, HDW and LYM, and the decrease in NEU observed in females treated with 2,000 mg/kg/day were statistically significant (P < 0.05) compared with the vehicle control group.

**Table 3:** Hematological values in rats orally treated with PM012

			Hematological values									
			
Parameter	Unit	Sex	0 mg/kg			500 mg/kg		1000 mg/kg		2000 mg/kg		
			
			4 Weeks	26 Weeks	Recovery	4 Weeks	26 Weeks	4 Weeks	26 Weeks	4 Weeks	26 Weeks	Recovery
WBC	10^3^/μL	Male	11.12 ± 2.81	8.37 ± 1.35	7.63 ± 2.04	14.14 ± 2.29	9.47 ± 2.14	11.17 ± 3.22	8.35 ± 2.56	10.88 ± 3.18	8.99 ± 1.97	7.70 ± 1.07
		Female	6.78 ± 0.78	4.73 ± 0.92	5.82 ± 1.86	7.61 ± 0.75	4.20 ± 0.95	9.15 ± 3.92	4.83 ± 1.50	8.89 ± 2.60	4.93 ± 1.06	5.65 ± 1.43
RBC	10^6^/μL	Male	8.03 ± 0.17	9.04 ± 0.28	8.72 ± 0.38	8.12 ± 0.24	9.12 ± 0.32	8.11 ± 0.20	8.86 ± 0.19	8.24 ± 0.06	8.89 ± 0.27	8.65 ± 0.26
		Female	7.99 ± 0.20	8.00 ± 0.25	7.68 ± 0.30	7.72 ± 0.43	8.09 ± 0.27	7.69 ± 0.29	8.12 ± 0.52	7.76 ± 0.18	7.96 ± 0.18	7.61 ± 0.22
HGB	g/dL	Male	15.38 ± 0.53	15.5 ± 0.3	15.0 ± 0.8	15.40 ± 0.26	15.8 ± 0.5	15.42 ± 0.50	15.2 ± 0.4	15.44 ± 0.33	15.2 ± 0.3	14.9 ± 0.5
		Female	14.86 ± 0.41	14.5 ± 0.5	13.7 ± 0.5	14.54 ± 0.52	14.5 ± 0.4	14.50 ± 0.69	14.6 ± 0.7	14.88 ± 0.41	14.4 ± 0.5	14.1 ± 0.4
HCT	%	Male	43.62 ± 1.40	46.2 ± 1.0	45.2 ± 2.4	43.62 ± 0.87	46.9 ± 1.3	43.76 ± 1.24	45.2 ± 1.4	43.82 ± 1.04	45.1 ± 0.9	44.3 ± 1.1
		Female	42.08 ± 0.88	42.5 ± 1.4	40.9 ± 1.4	41.50 ± 1.52	42.8 ± 1.4	40.94 ± 1.30	43.2 ± 1.8	41.74 ± 1.06	42.4 ± 1.2	41.5 ± 1.3
MCV	fL	Male	54.28 ± 1.10	51.1 ± 1.5	51.9 ± 2.5	53.72 ± 1.45	51.5 ± 1.0	54.00 ± 1.22	51.1 ± 1.1	53.14 ± 1.48	50.8 ± 1.5	51.2 ± 0.9
		Female	52.68 ± 0.31	53.1 ± 0.7	53.2 ± 1.0	53.82 ± 1.25	52.9 ± 1.8	53.28 ± 0.75	53.3 ± 2.3	53.84 ± 1.00	53.3 ± 1.2	54.7 ± 0.5
MCH	pg	Male	19.14 ± 0.43	17.2 ± 0.5	17.3 ± 0.8	19.00 ± 0.47	17.4 ± 0.4	19.06 ± 0.30	17.1 ± 0.3	18.74 ± 0.44	17.1 ± 0.4	17.2 ± 0.3
		Female	18.56 ± 0.19	18.1 ± 0.3	17.9 ± 0.2	18.88 ± 0.43	17.9 ± 0.5	18.88 ± 0.40	18.1 ± 0.7	19.18 ± 0.30	18.1 ± 0.5	18.4 ± 0.2
MCHC	g/dL	Male	35.22 ± 0.18	33.6 ± 0.4	33.3 ± 0.3	35.32 ± 0.57	33.7 ± 0.4	35.28 ± 0.41	33.6 ± 0.4	35.28 ± 0.47	33.7 ± 0.3	33.7 ± 0.4
		Female	35.24 ± 0.48	34.1 ± 0.5	33.7 ± 0.2	35.06 ± 0.29	33.9 ± 0.6	35.38 ± 0.90	33.9 ± 0.5	35.58 ± 0.26	34.0 ± 0.4	33.8 ± 0.2
RDW	%	Male	15.34 ± 0.47	12.3 ± 0.5	12.7 ± 0.6	15.30 ± 1.12	12.4 ± 0.5	15.52 ± 0.40	12.1 ± 0.4	15.24 ± 0.36	12.2 ± 0.3	12.5 ± 0.7
		Female	14.84 ± 0.38	11.1 ± 0.4	10.5 ± 0.4	13.96 ± 0.88	11.1 ± 0.5	14.58 ± 0.70	11.2 ± 0.5	14.18 ± 0.33	11.0 ± 0.3	11.0 ± 0.5
HDW	g/dL	Male	ND^a)^	2.60 ± 0.14	2.73 ± 0.21	ND^a)^	2.68 ± 0.13	ND^a)^	2.52 ± 0.18	ND^a)^	2.58 ± 0.14	2.53 ± 0.27
		Female	ND^a)^	2.43 ± 0.20	2.20 ± 0.11	ND^a)^	2.29 ± 0.18	ND^a)^	2.39 ± 0.24	ND^a)^	2.36 ± 0.20	2.52 ± 0.23
PLT	10^3^/μL	Male	945.2 ± 81.9	993.3 ± 82.7	10.54 ± 109.3	935.2 ± 37.8	1015.1 ± 107.4	869.6 ± 72.2	992.2 ± 66.3	930.4 ± 72.1	1024.1 ± 97.6	10.84.4 ± 77.0
		Female	997.0 ± 72.9	1016.5 ± 90.2	1013.2 ± 62.5	975.0 ± 43.6	1017.7 ± 69.6	997.8 ± 99.9	912.4 ± 264.7	985.6 ± 74.1	997.3 ± 91.4	1019.8 ± 79.7
MPV	fL	Male	5.83 ± 0.49	5.92 ± 0.27	5.94 ± 0.29	5.67 ± 0.22	5.94 ± 0.20	5.91 ± 0.29	5.82 ± 0.17	5.81 ± 0.20	5.78 ± 0.15	5.98 ± 0.37
		Female	5.90 ± 0.33	6.02 ± 0.13	5.88 ± 0.13	5.90 ± 0.09	6.16 ± 0.25	5.67 ± 0.19	7.12 ± 2.44	5.73 ± 0.15	6.36 ± 0.84	5.90 ± 0.19
RET	%	Male	2.2 ± 0.84	2.22 ± 0.22	2.31 ± 0.28	1.8 ± 0.84	2.20 ± 0.20	2.0 ± 1.41	2.10 ± 0.27	2.4 ± 1.14	2.15 ± 0.26	2.32 ± 0.30
		Female	3.4 ± 1.82	2.39 ± 0.33	1.81 ± 0.51	3.8 ± 1.30	2.47 ± 0.36	3.2 ± 1.48	2.30 ± 0.60	2.2 ± 1.64	2.26 ± 0.28	2.36 ± 0.27
NEU	%	Male	12.75 ± 3.77	26.6 ± 10.7	16.2 ± 4.5	9.81 ± 2.15	29.8 ± 8.9	14.30 ± 4.91	25.2 ± 11.0	13.06 ± 6.29	23.5 ± 7.1	17.5 ± 6.0
		Female	10.25 ± 1.99	16.8 ± 4.5	31.3 ± 13.9	9.06 ± 2.03	16.6 ± 2.3	9.87 ± 4.91	17.1 ± 5.3	9.46 ± 2.87	16.3 ± 3.6	15.4 ± 3.7
LYM	%	Male	79.76 ± 5.96	68.2 ± 10.7	77.2 ± 5.0	84.80 ± 2.56	64.6 ± 8.6	77.52 ± 5.76	69.0 ± 10.5	79.90 ± 7.43	70.6 ± 6.5	74.7 ± 7.8
		Female	82.46 ± 3.14	78.0 ± 4.5	61.9 ± 12.5	85.10 ± 2.67	77.6 ± 2.3	83.96 ± 5.54	76.4 ± 6.5	83.84 ± 2.46	77.8 ± 4.0	77.8 ± 4.1
MONO	%	Male	4.40 ± 1.85	3.05 ± 0.36	4.12 ± 1.52	2.77 ± 1.01	3.57 ± 1.09	4.41 ± 1.39	3.52 ± 0.96	3.66 ± 0.97	3.74 ± 0.90	5.36 ± 1.81
		Female	3.75 ± 1.08	2.77 ± 0.79	4.24 ± 1.05	2.84 ± 0.80	3.01 ± 0.65	2.90 ± 0.70	3.64 ± 0.98	3.33 ± 0.50	3.68 ± 0.94	3.92 ± 0.33
EOS	%	Male	0.64 ± 0.27	1.47 ± 0.36	1.52 ± 0.59	0.53 ± 0.32	1.29 ± 0.49	0.64 ± 0.10	1.62 ± 0.62	0.83 ± 0.39	1.40 ± 0.75	1.08 ± 0.42
		Female	0.95 ± 0.64	1.67 ± 0.47	1.74 ± 0.58	0.83 ± 0.33	2.04 ± 0.87	0.97 ± 0.52	1.79 ± 0.58	0.88 ± 0.29	1.36 ± 0.37	2.04 ± 0.42
BASO	%	Male	2.43 ± 0.75	0.12 ± 0.06	0.16 ± 0.05	2.10 ± 0.60	0.15 ± 0.05	3.11 ± 0.44	0.17 ± 0.08	2.56 ± 0.47	0.17 ± 0.07	0.22 ± 0.04
		Female	2.58 ± 0.61	0.12 ± 0.06	0.10 ± 0.00	2.18 ± 0.45	0.18 ± 0.15	2.31 ± 0.45	0.16 ± 0.08	2.50 ± 0.56	0.12 ± 0.04	0.12 ± 0.04
LUC	%	Male	ND^a)^	0.57 ± 0.22	0.78 ± 0.26	ND^a)^	0.63 ± 0.33	ND^a)^	0.58 ± 0.15	ND^a)^	0.61 ± 0.23	1.14 ± 0.92
		Female	ND^a)^	0.62 ± 0.21	0.74 ± 0.30	ND^a)^	0.65 ± 0.35	ND^a)^	0.86 ± 0.31	ND^a)^	0.72 ± 0.35	0.74 ± 0.28

* WBC: white blood cell, RBC: red blood cell, HGB: hemoglobin concentration, HCT: hematocrit, MCV: mean corpuscular volume, MCH: mean corpuscular hemoglobin, MCHC: mean corpuscular hemoglobin concentration, RDW: Red cell distribution width, PLT: Platelet, MPV: Mean platelet volume, RET: Reticulocyte, NEU: Neutrophil, LYM: Lymphocyte, MONO: Monocyte, EOS: Eosinophil, BASO: Basophil. Values are mean ± SD (n = 5 (4 weeks) or 10 (26 weks)/sex/dose). P < 0.05 compare with normal group. ^b) ^ND, not determined.

#### Serum biochemistry

Serum biochemistry data are summarized in Table 4. There were no significant changes in the levels of CPK, GLU, CHO, TG, A/G, BUN, CRE or IP. Female mice treated with 2,000 mg/kg/day PM012 extract exhibited significantly increased PRO. No statistically significant differences in serum electrolytes such as K^+ ^or Cl^- ^were noted. Male mice treated with 1,000 mg/kg/day PM012 extract exhibited significantly decreased Ca^2+ ^(main groups). Moreover, female mice treated with 1,000 mg/kg/day PM012 extract exhibited significantly decreased Na^+ ^(main groups). The effects of PM012 on liver function parameters such as AST, ALT, ALP, and BIL in serum were also investigated. Female mice treated with 500 and 2,000 mg/kg/day PM012 extract exhibited significantly increased ALB. The 1,000 mg/kg/day exposure groups and recovery groups, however, did not exhibit significant changes in serum AST, ALT, ALP or BIL.

**Table 4:** Serum biochemical values in rats orally treated with PM012

			Dose (mg/kg)									
			
Parameter	Units	Sex	0			500		1000		2000		
			
			4 Weeks	26 Weeks	Recovery	4 Weeks	26 Weeks	4 Weeks	26 Weeks	4 Weeks	26 Weeks	Recovery
AST	U/L	Male	113.90 ± 12.59	83.1 ± 11.4	90.0 ± 12.8	101.98 ± 14.09	78.5 ± 9.5	94.46 ± 12.02	85.9 ± 14.5	108.66 ± 24.97	84.1 ± 12.7	85.1 ± 21.5
		Female	97.10 ± 12.99	82.0 ± 10.0	83.9 ± 17.6	102.54 ± 16.78	81.9 ± 16.5	102.88 ± 7.01	81.9 ± 16.6	107.64 ± 21.81	80.3 ± 9.7	81.3 ± 13.2
ALT	U/L	Male	35.06 ± 4.56	50.3 ± 10.5	52.1 ± 10.3	34.10 ± 4.28	44.3 ± 6.6	35.56 ± 4.18	48.8 ± 12.9	34.68 ± 4.66	41.9 ± 6.7	52.6 ± 8.4
		Female	27.14 ± 3.54	37.3 ± 7.8	36.4 ± 3.6	25.96 ± 2.36	35.8 ± 3.7	27.64 ± 1.90	33.4 ± 5.6	27.06 ± 1.64	36.1 ± 6.5	35.2 ± 3.9
ALP	U/L	Male	133.96 ± 13.75	76.7 ± 11.9	84.6 ± 11.9	124.38 ± 9.96	77.0 ± 11.0	126.12 ± 9.52	79.5 ± 14.9	147.08 ± 19.36	73.1 ± 12.2	91.1 ± 9.1
		Female	87.20 ± 10.59	57.9 ± 12.2	54.1 ± 8.7	92.66 ± 15.55	62.3 ± 28.8	99.04 ± 18.32	50.6 ± 16.5	88.48 ± 3.48	54.7 ± 11.5	58.4 ± 13.4
CPK	U/L	Male	408.4 ± 68.2	101.6 ± 25.6	199.8 ± 118.8	291.6 ± 96.3	98.9 ± 20.5	212.4 ± 92.1	114.7 ± 49.5	261.0 ± 152.6	122.8 ± 65.9	135.2 ± 64.9
		Female	265.8 ± 110.2	104.1 ± 31.7	143.8 ± 70.5	266.6 ± 142.2	87.2 ± 45.2	227.8 ± 78.6	81.9 ± 28.9	282.0 ± 148.6	85.0 ± 33.1	161.2 ± 68.4
BIL	mg/dL	Male	0.21 ± 0.00	0.19 ± 0.02	0.19 ± 0.02	0.21 ± 0.00	0.21 ± 0.02	0.20 ± 0.01	0.19 ± 0.01	0.21 ± 0.01	0.20 ± 0.02	0.20 ± 0.01
		Female	0.21 ± 0.02	0.23 ± 0.03	0.26 ± 0.04	0.23 ± 0.02	0.23 ± 0.03	0.22 ± 0.02	0.23 ± 0.03	0.23 ± 0.02	0.24 ± 0.02	0.26 ± 0.05
GLU	mg/dL	Male	102.18 ± 9.12	116.8 ± 9.1	120.5 ± 9.5	99.94 ± 5.77	121.3 ± 12.3	108.70 ± 9.87	113.7 ± 11.4	109.38 ± 8.60	113.3 ± 9.8	121.0 ± 8.4
		Female	106.66 ± 7.29	107.6 ± 10.7	130.5 ± 14.1	99.86 ± 3.54	111.6 ± 15.7	100.10 ± 5.58	110.0 ± 11.2	99.30 ± 9.04	106.2 ± 12.2	132.0 ± 7.4
CHO	mg/dL	Male	90.4 ± 6.6	139.5 ± 27.3	165.0 ± 46.6	93.2 ± 12.8	152.9 ± 46.8	95.6 ± 9.4	123.2 ± 26.6	84.6 ± 9.9	116.0 ± 25.2	180.2 ± 73.0
		Female	96.8 ± 13.2	118.9 ± 13.0	119.0 ± 20.9	96.4 ± 20.6	119.1 ± 33.0	98.0 ± 11.6	138.5 ± 34.5	98.2 ± 17.2	126.4 ± 28.2	131.4 ± 24.5
TG	mg/dL	Male	45.2 ± 8.2	49.6 ± 8.3	54.8 ± 18.9	46.8 ± 11.1	61.1 ± 20.2	44.4 ± 7.1	46.0 ± 10.4	46.4 ± 7.4	47.7 ± 7.3	63.4 ± 34.0
		Female	36.0 ± 8.1	43.5 ± 5.5	39.6 ± 5.3	36.4 ± 7.7	43.2 ± 6.6	36.2 ± 4.1	48.1 ± 6.5	39.4 ± 6.5	47.6 ± 9.0	44.0 ± 13.1
PRO	g/dL	Male	6.04 ± 0.10	6.54 ± 0.14	6.38 ± 0.17	5.99 ± 0.23	6.48 ± 0.19	5.97 ± 0.10	6.42 ± 0.13	5.99 ± 0.24	6.50 ± 0.17	6.60 ± 0.21
		Female	5.63 ± 0.23	6.37 ± 0.16	6.84 ± 0.33	5.81 ± 0.38	6.65 ± 0.35	5.71 ± 0.23	6.60 ± 0.30	5.79 ± 0.13	6.76 ± 0.32	6.53 ± 0.20
ALB	g/dL	Male	3.17 ± 0.05	2.99 ± 0.10	2.85 ± 0.14	3.14 ± 0.08	2.95 ± 0.11	3.15 ± 0.04	2.97 ± 0.07	3.18 ± 0.11	3.07 ± 0.12	2.83 ± 0.21
		Female	3.05 ± 0.11	3.16 ± 0.10	3.44 ± 0.26	3.16 ± 0.12	3.34 ± 0.12	3.08 ± 0.09	3.27 ± 0.19	3.14 ± 0.04	3.38 ± 0.20	3.25 ± 0.16
A/G	ratio	Male	1.10 ± 0.00	0.84 ± 0.03	0.81 ± 0.09	1.10 ± 0.04	0.84 ± 0.08	1.12 ± 0.02	0.86 ± 0.05	1.14 ± 0.03	0.90 ± 0.06	0.76 ± 0.11
		Female	1.19 ± 0.05	0.98 ± 0.06	1.01 ± 0.10	1.20 ± 0.10	1.01 ± 0.05	1.17 ± 0.05	0.98 ± 0.05	1.18 ± 0.05	1.00 ± 0.05	0.99 ± 0.05
BUN	mg/dL	Male	16.94 ± 1.66	16.0 ± 2.7	17.1 ± 3.5	15.26 ± 1.69	16.4 ± 2.1	14.52 ± 1.38	15.8 ± 2.3	15.86 ± 1.88	14.7 ± 2.4	16.9 ± 2.9
		Female	16.48 ± 1.47	17.5 ± 1.2	15.2 ± 2.0	16.60 ± 1.02	17.3 ± 1.1	16.24 ± 1.39	17.7 ± 1.9	17.14 ± 0.66	16.9 ± 1.9	14.6 ± 1.5
CRE	mg/dL	Male	0.48 ± 0.07	0.57 ± 0.08	0.59 ± 0.07	0.49 ± 0.03	0.60 ± 0.04	0.48 ± 0.02	0.56 ± 0.03	0.50 ± 0.02	0.56 ± 0.02	0.62 ± 0.07
		Female	0.50 ± 0.03	0.63 ± 0.03	0.62 ± 0.04	0.52 ± 0.04	0.64 ± 0.05	0.53 ± 0.05	0.68 ± 0.07	0.53 ± 0.03	0.65 ± 0.05	0.61 ± 0.02
IP	mg/dL	Male	8.57 ± 0.35	5.96 ± 0.38	5.92 ± 0.53	8.59 ± 0.14	6.16 ± 0.36	8.43 ± 0.42	6.08 ± 0.44	8.76 ± 0.39	6.37 ± 0.59	6.13 ± 0.14
		Female	8.08 ± 0.62	5.69 ± 0.86	4.43 ± 0.28	7.96 ± 0.58	5.17 ± 1.28	8.08 ± 0.50	5.53 ± 0.71	7.95 ± 0.38	5.76 ± 0.79	4.68 ± 0.24
Ca^2+^	mg/dL	Male	10.53 ± 0.14	10.48 ± 0.15	10.30 ± 0.28	10.47 ± 0.21	10.41 ± 0.20	10.40 ± 0.18	10.26 ± 0.09	10.34 ± 0.25	10.32 ± 0.13	10.39 ± 0.05
		Female	10.08 ± 0.25	10.08 ± 0.21	10.48 ± 0.34	10.05 ± 0.22	10.19 ± 0.31	10.03 ± 0.32	10.13 ± 0.22	9.81 ± 0.18	10.20 ± 0.22	10.21 ± 0.28
Na^+^	mmol/L	Male	148.2 ± 0.8	145.3 ± 0.9	143.9 ± 1.2	148.2 ± 0.8	144.7 ± 1.0	148.2 ± 1.3	144.5 ± 0.8	148.6 ± 0.9	145.3 ± 1.3	143.2 ± 0.7
		Female	146.2 ± 1.3	145.0 ± 0.9	141.9 ± 1.1	145.2 ± 2.4	144.1 ± 1.2	143.8 ± 0.8	143.5 ± 1.3	144.0 ± 1.0	143.5 ± 0.9	142.0 ± 1.1
K^+^	mmol/L	Male	4.82 ± 0.13	4.32 ± 0.18	4.57 ± 0.23	4.71 ± 0.22	4.19 ± 0.17	4.75 ± 0.12	4.36 ± 0.20	4.72 ± 0.14	4.31 ± 0.21	4.37 ± 0.25
		Female	4.71 ± 0.16	3.99 ± 0.15	3.99 ± 0.24	4.52 ± 0.16	4.36 ± 1.08	4.53 ± 0.11	4.20 ± 1.08	4.53 ± 0.43	4.04 ± 0.29	3.96 ± 0.32
Cl^-^	mmol/L	Male	108.8 ± 0.4	105.7 ± 0.7	104.5 ± 1.2	108.4 ± 1.1	105.0 ± 1.8	108.2 ± 0.8	106.1 ± 0.9	108.6 ± 0.5	105.2 ± 1.1	103.5 ± 1.0
		Female	109.2 ± 0.8	106.5 ± 1.4	104.6 ± 0.9	109.2 ± 0.8	107.1 ± 0.7	108.0 ± 1.2	106.4 ± 0.9	108.2 ± 0.8	105.9 ± 1.1	104.7 ± 1.5

*AST (Aspartate aminotransferase), ALT (Alanine aminotransferase), ALP (Alkaline phosphatase), CPK (Creatine phosphokinase), BIL (Total bilirubin), GLU (Glucose), CHO (Total cholesterol), TG (Triglyceride), PRO (Total protein), ALB (Albumin), A/G ratio (Albumin/Globulin ratio), BUN (Blood urea nitrogen), CRE (Creatinine), IP (Inorganic phosphorus), Ca^2+ ^(Calcium ion), Na^+ ^(Sodium ion), K^+ ^(Potassium ion), Cl^- ^(Chloride ion).

Values are mean ± SD (n = 5 (4 weeks) or 10 (26 weks)/sex/dose). P < 0.05 compare with normal group.

#### Organ weights

Organ weight data are summarized in Table 5. Thymus weight showed a dose-related increasing trend in the main and recovery female groups and a significant increase (P < 0.01) was identified in the 2,000 mg/kg/day recovery group. No statistically significantly differences were observed in males

**Table 5:** Absolute & relative organ weights in rats orally treated with PM012

		Dose (mg/kg)									
		
Parameter	Sex	0			500		1000		2000		
		
		4 Weeks	26 Weeks	Recovery	4 Weeks	26 Weeks	4 Weeks	26 Weeks	4 Weeks	26 Weeks	Recovery
Body weight^a)^	Male	326.79 ± 18.46	484.71 ± 33.01	502.94 ± 17.59	321.28 ± 20.83	504.38 ± 45.11	330.67 ± 23.75	474.08 ± 46.03	324.80 ± 15.32	468.68 ± 35.08	507.84 ± 32.08
	Female	194.35 ± 7.86	268.94 ± 12.88	277.90 ± 26.72	195.07 ± 9.64	270.36 ± 13.17	196.26 ± 12.33	272.81 ± 19.48	195.04 ± 14.05	264.92 ± 17.98	279.89 ± 6.03
Adrenal grand (Lt.)	Male	0.0266 ± 0.0032	0.0258 ± 0.0025	0.0276 ± 0.0037	0.0260 ± 0.0033	0.0254 ± 0.0031	0.0265 ± 0.0025	0.0245 ± 0.0022	0.0290 ± 0.0011	0.0243 ± 0.0031	0.0271 ± 0.0037
	Female	0.0312 ± 0.0025	0.0311 ± 0.0038	0.0309 ± 0.0054	0.0309 ± 0.0023	0.0325 ± 0.0039	0.0333 ± 0.0025	0.0313 ± 0.0056	0.0314 ± 0.0038	0.0310 ± 0.0050	0.0326 ± 0.0053
% to body weight	Male	0.0082 ± 0.0014	0.0054 ± 0.0007	0.0055 ± 0.0007	0.0081 ± 0.0009	0.0050 ± 0.0005	0.0080 ± 0.0005	0.0052 ± 0.0006	0.0089 ± 0.0007	0.0052 ± 0.0006	0.0053 ± 0.0005
	Female	0.0161 ± 0.0016	0.0115 ± 0.0013	0.0112 ± 0.0022	0.0158 ± 0.0008	0.0120 ± 0.0013	0.0170 ± 0.0010	0.0115 ± 0.0019	0.0162 ± 0.0023	0.0117 ± 0.0020	0.0117 ± 0.0019
Adrenal grand (Rt.)	Male	0.0274 ± 0.0015	0.0247 ± 0.0027	0.0289 ± 0.0025	0.0255 ± 0.0015	0.0252 ± 0.0030	0.0246 ± 0.0046	0.0224 ± 0.0034	0.0264 ± 0.0026	0.0246 ± 0.0026	0.0278 ± 0.0054
	Female	0.0305 ± 0.0023	0.0295 ± 0.0030	0.0296 ± 0.0059	0.0308 ± 0.0045	0.0306 ± 0.0033	0.0305 ± 0.0019	0.0301 ± 0.0052	0.0313 ± 0.0041	00287 ± 0.0042	0.0314 ± 0.0048
% to body weight	Male	0.0084 ± 0.0008	0.0051 ± 0.0006	0.0057 ± 0.0005	0.0080 ± 0.0003	0.0050 ± 0.0003	0.0074 ± 0.0012	0.0048 ± 0.0009	0.0081 ± 0.0009	0.0053 ± 0.0006	0.0055 ± 0.0009
	Female	0.0157 ± 0.0013	0.0110 ± 0.0013	0.0108 ± 0.0026	0.0158 ± 0.0020	0.0113 ± 0.0010	0.0156 ± 0.0017	0.0110 ± 0.0019	0.0160 ± 0.0019	0.0109 ± 0.0018	0.0112 ± 0.0018
Pituitary grand	Male	ND^b)^	0.0128 ± 0.0010	0.0135 ± 0.0017	ND^b)^	0.0126 ± 0.0011	ND^b)^	0.0126 ± 0.0013	ND^b)^	0.0124 ± 0.0009	0.0135 ± 0.0014
	Female	ND^b)^	0.0151 ± 0.0017	0.0153 ± 0.0011	ND^b)^	0.0150 ± 0.0022	ND^b)^	0.0170 ± 0.0042	ND^b)^	0.0143 ± 0.0021	0.0160 ± 0.0017
% to body weight	Male	ND^b)^	0.0026 ± 0.0002	0.0027 ± 0.0004	ND^b)^	0.0025 ± 0.0002	ND^b)^	0.0027 ± 0.0002	ND^b)^	0.0027 ± 0.0001	0.0027 ± 0.0002
	Female	ND^b)^	0.0056 ± 0.0005	0.0056 ± 0.0007	ND^b)^	0.0055 ± 0.0009	ND^b)^	0.0062 ± 0.0015	ND^b)^	0.0054 ± 0.0009	0.0057 ± 0.0005
Thymus	Male	0.4132 ± 0.0579	0.1887 ± 0.0403	0.1668 ± 0.0116	0.5040 ± 0.0841	0.2201 ± 0.0349	0.4522 ± 0.0657	0.2055 ± 0.0585	0.4942 ± 0.0904	0.1981 ± 0.0361	0.1520 ± 0.0308
	Female	0.2837 ± 0.0195	0.1380 ± 0.0191	0.1320 ± 0.0144	0.3389 ± 0.0336	0.1418 ± 0.0126	0.3124 ± 0.0420	0.1535 ± 0.0347	0.3372 ± 0.0316	0.1533 ± 0.0233	0.1748 ± 0.0180**
% to body weight	Male	0.1269 ± 0.0197	0.0388 ± 0.0074	0.0332 ± 0.0022	0.1583 ± 0.0338	0.0437 ± 0.0059	0.1378 ± 0.0261	0.0433 ± 0.0111	0.1515 ± 0.0229	0.0424 ± 0.0076	0.0302 ± 0.0073
	Female	0.1462 ± 0.0121	0.0515 ± 0.0083	0.0478 ± 0.0070	0.1739 ± 0.0168**	0.0527 ± 0.0063	0.1588 ± 0.0146	0.0563 ± 0.0126	0.1729 ± 0.0095**	0.0580 ± 0.0088	0.0624 ± 0.0057**
Prostate	Male	0.2664 ± 0.0587	0.5802 ± 0.1913	0.5616 ± 0.1757	0.3106 ± 0.1454	0.6464 ± 0.1822	0.3022 ± 0.1208	0.5570 ± 0.1379	0.2706 ± 0.0913	0.6609 ± 0.1472	0.5776 ± 0.0680
% to body weight		0.0811 ± 0.0150	0.1215 ± 0.0438	0.1109 ± 0.0312	0.0950 ± 0.0412	0.1276 ± 0.0318	0.0921 ± 0.0389	0.1181 ± 0.0294	0.0825 ± 0.0256	0.1408 ± 0.0278	0.1141 ± 0.0152
Testis (Lt.)	Male	1.8696 ± 0.0630	2.0628 ± 0.1010	2.2032 ± 0.0711	1.8118 ± 0.2120	2.1428 ± 0.1616	1.7334 ± 0.2977	1.9960 ± 0.1191	1.8244 ± 0.0591	2.1109 ± 0.1282	2.0692 ± 0.1961
% to body weight		0.5739 ± 0.0444	0.4272 ± 0.0342	0.4385 ± 0.0211	0.5628 ± 0.0395	0.4264 ± 0.0331	0.5235 ± 0.0758	0.4242 ± 0.0432	0.5628 ± 0.0349	0.4522 ± 0.0376	0.4075 ± 0.0315
Testis (Rt.)	Male	1.8672 ± 0.0834	2.0352 ± 0.1580	2.1622 ± 0.0698	1.8232 ± 0.2055	2.1153 ± 0.1467	1.7288 ± 0.2737	2.0206 ± 0.1294	1.8008 ± 0.0563	2.0990 ± 0.1063	2.0372 ± 0.1672
% to body weight		0.5734 ± 0.0512	0.4218 ± 0.0453	0.4304 ± 0.0209	0.5664 ± 0.0357	0.4215 ± 0.0376	0.5226 ± 0.0718	0.4302 ± 0.0524	0.5553 ± 0.0288	0.4500 ± 0.0388	0.4021 ± 0.0375
Epididymis (Lt.)	Male	ND^b)^	0.7142 ± 0.0282	0.7672 ± 0.0424	ND^b)^	0.7532 ± 0.0594	ND^b)^	0.7185 ± 0.0632	ND^b)^	0.7396 ± 0.0401	0.7240 ± 0.0396
% to body weight		ND^b)^	0.1478 ± 0.0092	0.1529 ± 0.0126	ND^b)^	0.1499 ± 0.0120	ND^b)^	0.1526 ± 0.0181	ND^b)^	0.1584 ± 0.0120	0.1429 ± 0.0097
Epididymis (Rt.)	Male	ND^b)^	0.7352 ± 0.0372	0.7620 ± 0.0299	ND^b)^	0.7572 ± 0.0604	ND^b)^	0.7429 ± 0.0493	ND^b)^	0.7501 ± 0.0461	0.7184 ± 0.0322
% to body weight		ND^b)^	0.1523 ± 0.0131	0.1517 ± 0.0090	ND^b)^	0.1507 ± 0.0121	ND^b)^	0.1578 ± 0.0157	ND^b)^	0.1606 ± 0.0127	0.1417 ± 0.0062
Ovary (Lt.)	Female	0.0443 ± 0.0089	0.0388 ± 0.0120	0.0302 ± 0.0085	0.0434 ± 0.0037	0.0397 ± 0.0093	0.0452 ± 0.0077	0.0354 ± 0.0066	0.0411 ± 0.0051	0.0403 ± 0.0132	0.0317 ± 00129
% to body weight		0.0227 ± 0.0040	0.0143 ± 0.0040	0.0108 ± 0.0023	0.0223 ± 0.0024	0.0146 ± 0.0031	0.0230 ± 0.0035	0.0131 ± 0.0033	0.0210 ± 0.0020	0.0151 ± 0.0044	0.0114 ± 0.0048
Ovary (Rt.)	Female	0.0453 ± 0.0047	0.0375 ± 0.0087	0.0305 ± 0.0134	0.0430 ± 0.0067	0.0413 ± 0.0102	0.0454 ± 0.0081	0.0346 ± 0.0079	0.0456 ± 0.0071	0.0360 ± 0.0105	0.0296 ± 0.0078
% to body weight		0.0233 ± 0.0023	0.0139 ± 0.0031	0.0107 ± 0.0035	0.0221 ± 0.0036	0.0153 ± 0.0037	0.0231 ± 0.0035	0.0127 ± 0.0031	0.0234 ± 0.0035	0.0135 ± 0.0034	0.0106 ± 0.0029
Uterus	Female	0.6020 ± 0.5041	0.7540 ± 0.2803	1.5136 ± 1.7081	0.7301 ± 0.3711	0.7690 ± 0.2454	0.4385 ± 0.1144	0.6901 ± 0.2825	0.4302 ± 0.1316	1.0180 ± 0.7969	0.7328 ± 0.1301
% to body weight		0.3134 ± 0.2721	0.2805 ± 0.1043	0.5421 ± 0.5875	0.3756 ± 0.1879	0.2866 ± 0.0986	0.2219 ± 0.0471	0.2506 ± 0.0902	0.2244 ± 0.0825	0.3884 ± 0.3038	0.2615 ± 0.0441
Spleen	Male	0.7080 ± 0.0403	0.8423 ± 0.1620	0.9244 ± 0.1689	0.7766 ± 0.0442	0.8627 ± 0.1149	0.8082 ± 0.1229	0.8115 ± 0.1162	0.6982 ± 0.1578	0.8396 ± 0.0962	0.9108 ± 0.2680
	Female	0.5174 ± 0.0475	0.6023 ± 0.0677	0.5912 ± 0.0969	0.5178 ± 0.0523	0.5904 ± 0.0496	0.5450 ± 0.0515	0.5846 ± 0.0497	0.5438 ± 0.0540	0.5765 ± 0.0545	0.6338 ± 0.0327
% to body weight	Male	0.2167 ± 0.0048	0.1730 ± 0.0254	0.1838 ± 0.0339	0.2427 ± 0.0232	0.1712 ± 0.0202	0.2436 ± 0.0258	0.1712 ± 0.0173	0.2138 ± 0.0418	0.1796 ± 0.0205	0.1781 ± 0.0461
	Female	0.2665 ± 0.0256	0.2239 ± 0.0219	0.2117 ± 0.0160	0.2655 ± 0.0242	0.2182 ± 0.0115	0.2778 ± 0.0218	0.2152 ± 0.0231	0.2786 ± 0.0136	0.2177 ± 0.0161	0.2264 ± 0.0087
Kidney (Lt.)	Male	1.0834 ± 0.0737	1.4412 ± 0.0916	1.5350 ± 0.1345	1.1088 ± 0.1901	1.4725 ± 0.2157	1.1310 ± 0.0947	1.3753 ± 0.1792	1.0716 ± 0.0701	1.4461 ± 0.3356	1.4692 ± 0.2390
	Female	0.6434 ± 0.0440	0.7791 ± 0.0439	0.8626 ± 0.0847	0.6320 ± 0.0439	0.7969 ± 0.0477	0.6650 ± 0.0495	0.8142 ± 0.0688	0.6674 ± 0.0675	0.8092 ± 0.0705	0.8742 ± 0.0311
% to body weight	Male	0.3318 ± 0.0193	0.2978 ± 0.0159	0.3050 ± 0.0219	0.3432 ± 0.0376	0.2910 ± 0.0249	0.3419 ± 0.0083	0.2899 ± 0.0205	0.3299 ± 0.0131	0.3073 ± 0.0569	0.2890 ± 0.0406
	Female	0.3312 ± 0.0210	0.2901 ± 0.0188	0.3113 ± 0.0296	0.3238 ± 0.0086	0.2949 ± 0.0128	0.3387 ± 0.0089	0.2988 ± 0.0206	0.3417 ± 0.0114	0.3057 ± 0.0212	0.3124 ± 0.0119
Kidney (Rt.)	Male	1.0920 ± 0.0966	1.4783 ± 0.0904	1.5424 ± 0.1388	1.0810 ± 0.1098	1.5046 ± 0.2471	1.1354 ± 0.0658	1.3637 ± 0.1638	1.0760 ± 0.0876	1.3921 ± 0.1399	1.4910 ± 0.2666
	Female	0.6700 ± 0.0245	0.7974 ± 0.0511	0.8416 ± 0.0896	0.6542 ± 0.0316	0.8195 ± 0.0634	0.6956 ± 0.0681	0.8177 ± 0.0871	0.6604 ± 0.0610	0.8213 ± 0.0652	0.8740 ± 0.0535
% to body weight	Male	0.3345 ± 0.0286	0.3055 ± 0.0175	0.3064 ± 0.0217	0.3361 ± 0.0184	0.2969 ± 0.0260	0.3437 ± 0.0108	0.2879 ± 0.0218	0.3310 ± 0.0159	0.2969 ± 0.0165	0.2931 ± 0.0453
	Female	0.3451 ± 0.0156	0.2967 ± 0.0179	0.3030 ± 0.0199	0.3355 ± 0.0096	0.3032 ± 0.0199	0.3539 ± 0.0160	0.2997 ± 0.0257	0.3382 ± 0.0090	0.3104 ± 0.0204	0.3125 ± 0.0224
Heart	Male	1.1572 ± 0.0898	1.6088 ± 0.1665	1.5266 ± 0.1471	1.1302 ± 0.2075	1.5934 ± 0.1625	1.1730 ± 0.0850	1.4677 ± 0.1040	1.1562 ± 0.0871	1.5236 ± 0.1646	1.5334 ± 0.2367
	Female	0.7574 ± 0.0447	0.9366 ± 0.0652	0.9298 ± 0.1196	0.7590 ± 0.0380	0.9729 ± 0.0742	0.7254 ± 0.0599	0.9469 ± 0.0530	0.7158 ± 0.0575	1.0124 ± 0.3391	0.9704 ± 0.0845
% to body weight	Male	0.3543 ± 0.0229	0.3317 ± 0.0228	0.3034 ± 0.0256	0.3499 ± 0.0445	0.3160 ± 0.0185	0.3551 ± 0.0177	0.3118 ± 0.0324	0.3560 ± 0.0208	0.3250 ± 0.0230	0.3010 ± 0.0324
	Female	0.3896 ± 0.0119	0.3484 ± 0.0209	0.3344 ± 0.0242	0.3893 ± 0.0152	0.3601 ± 0.0259	0.3705 ± 0.0346	0.3477 ± 0.0142	0.3683 ± 0.0374	0.3845 ± 0.1360	0.3465 ± 0.0266
Lung	Male	1.5656 ± 0.0698	1.9947 ± 0.1779	2.0066 ± 0.1561	1.4874 ± 0.1229	2.0049 ± 0.1510	1.5250 ± 0.1239	1.8534 ± 0.1209	1.5416 ± 0.1092	1.9613 ± 0.1305	1.8042 ± 0.1591
	Female	1.1890 ± 0.0432	1.4361 ± 0.1088	1.4584 ± 0.1603	1.2182 ± 0.1431	1.4594 ± 0.0892	1.2164 ± 0.1588	1.4234 ± 0.0721	1.1466 ± 0.0518	1.4478 ± 0.1077	1.4262 ± 0.1322
% to body weight	Male	0.4797 ± 0.0204	0.4114 ± 0.0208	0.3987 ± 0.0234	0.4637 ± 0.0366	0.3982 ± 0.0178	0.4610 ± 0.0128	0.3923 ± 0.0204	0.4744 ± 0.0180	0.4205 ± 0.0415	0.3571 ± 0.0440
	Female	0.6121 ± 0.0203	0.5337 ± 0.0229	0.5264 ± 0.0574	0.6234 ± 0.0522	0.5403 ± 0.0305	0.6207 ± 0.0833	0.5233 ± 0.0339	0.5891 ± 0.0283	0.5469 ± 0.0270	0.5094 ± 0.0439
Brain	Male	ND^b)^	2.0641 ± 0.0562	2.0344 ± 0.0501	NDb)	2.0215 ± 0.0849	NDb)	1.9862 ± 0.0473	NDb)	2.0204 ± 0.0423	2.0450 ± 0.0802
	Female	ND^b)^	1.8062 ± 0.0368	1.8284 ± 0.0730	NDb)	1.8646 ± 0.0732	NDb)	1.8016 ± 0.0678	NDb)	1.8069 ± 0.0602	1.8722 ± 0.0673
% to body weight	Male	ND^b)^	0.4275 ± 0.0298	0.4048 ± 0.0137	NDb)	0.4026 ± 0.0255	NDb)	0.4220 ± 0.0351	NDb)	0.4329 ± 0.0277	0.4042 ± 0.0335
	Female	ND^b)^	0.6726 ± 0.0258	0.6629 ± 0.0681	NDb)	0.6902 ± 0.0214	NDb)	0.6628 ± 0.0431	NDb)	0.6841 ± 0.0382	0.6695 ± 0.0368
Liver	Male	9.5912 ± 0.7911	11.8528 ± 1.1643	12.6104 ± 1.7035	9.3626 ± 0.9629	12.5935 ± 2.2636	10.1466 ± 0.5700	11.1793 ± 1.7555	9.5052 ± 0.5582	11.0740 ± 0.9159	13.0788 ± 2.8997
	Female	5.5920 ± 0.5099	6.4589 ± 0.7810	6.4812 ± 0.6049	5.5784 ± 0.5334	6.8322 ± 1.1330	5.5102 ± 0.3767	6.8610 ± 1.7458	5.5738 ± 0.5615	6.6866 ± 0.5558	6.6626 ± 0.9795
% to body weight	Male	2.9356 ± 0.1935	2.4422 ± 0.1178	2.5046 ± 0.3008	2.9083 ± 0.1159	2.4815 ± 0.2634	3.0727 ± 0.1101	2.3528 ± 0.2271	2.9256 ± 0.0668	2.3638 ± 0.1116	2.5611 ± 0.4535
	Female	2.8812 ± 0.2921	2.3981 ± 0.2116	2.3332 ± 0.0680	2.8595 ± 0.2334	2.5241 ± 0.3756	2.8115 ± 0.1862	2.4970 ± 0.4764	2.8554 ± 0.1547	2.5322 ± 0.2457	2.3763 ± 0.3002

^a) ^Represents body weights right before necropsy, after fasting.

^b) ^ND, not determined.

Values are mean ± SD (n = 5 (4 weeks or recovery) or 10 (26 weks)/sex/dose). P < 0.05 compare with normal group.

#### Histopathology

Histopathological examinations are an important aspect of safety assessments. A macroscopic examination of vital organs found no abnormalities. Histological evaluation of the adrenal gland, pituitary gland, prostate gland, kidney, liver, spleen, lung, heart, thymus, thyroid gland, mesenteric and harderian glands did not reveal any pathological changes in the highest dose group (data not shown).

## Discussion

Natural products have long been used in traditional medicine to treat various diseases. Extracts prepared from medicinal plants and other natural sources contain a variety of molecules with potent biological activities. However, it is often difficult to analyze the biological activities of these extracts because of their complex nature and the possible synergistic effects of their components. Recently, the raw materials of such products have been used to develop new drugs [1,25,26].

Many studies have reported the pharmacological efficacy of PM012. PM012 was shown to have potent protection effects against ibotenic acid-induced cell damage and cognitive deficits in a previous study [27]. In addition, PM012 treatment accelerated the speed of information processing and enhanced cognitive abilities in normal subjects [28]. PM012 may repair cognitive impairment induced by dementia through an increase of acetylcholine synthesis in combination with the other advantageous effects mentioned above, thus helping to alleviate the symptoms of dementia patients [15,27,28].

The components of PM012 have been shown to have various effects when used individually. *Mountain cortex radicis*, a component of PM012, decreased ROS generation and cytotoxicity in hydrogen peroxide-stimulated neuronal cells through increasing the expression of the genes heme oxygenase and COMT, which play a major role in regulating ROS production [29]. *Rehmanniae Radix*, another component of PM012, improved learning and memory in monosodium glutamate-treated rats through anti-oxidation and increased the expression of hippocampal c-fos and NGF and intelligence in humans [2,3]. It has been reported that *Lycii Fructus *and *Corni fructus*, additional components of PM012, have strong anti-oxidative effects [4,30]. Furthermore, *Lycii Fructus *[31], *Corni fructus *[32], *Rehmannia radix *[21], *Discoreae radix *[33], and *Mountain cortex radicis *[34], have all shown anti-inflammatory effects. However, to date, there is no information on any aspects, such as oral toxicity, of the safety of any of these components or PM012. Therefore, we investigated and here report the safety of repeated oral administration as determined by a toxicity experiment conducted with a crude extract of PM012 using male and female SD rats.

The present study was performed to evaluate the efficacy of PM012 in hPS2m transgenic mice and the toxicity of PM012 in SD rats after 4- or 26-week repeated oral administrations and to identify the no observed adverse effect level (NOAEL), target organs and the recovery potential during the 4-week recovery study. The Morris water maze task was used to test the effect of PM012 on the relative spatial learning capability and reference memory in hPS2m mice to determine whether PM012 can protect against memory impairment. The Morris water maze spatial learning task has been used in the validation of rodent models for neurocognitive disorders and for the evaluation of possible neurocognitive treatments. In the current study, the latency to find the platform in acquisition trials by the PM012-treated group was significantly decreased compared to the untreated group. The PM012-treated group also spent a greater proportion of the probe trial searching in the training quadrant, demonstrating that PM012 attenuated the hPS2 mutation-induced learning and memory deficits. Weight loss is a frequent condition of AD, occurring in about 30%-40% of all AD patients [35,36]. Body weight was significantly increased in the PM012-treated group, suggesting possible beneficial effects of PM012 on AD progression. In terms of safety, no mortality was observed and no abnormal changes were observed in the body weight, food and water consumption, ophthalmic examination, urinalysis and blood clotting time test.

In the clinical examination of the rats used in the safety assessment, some rats presented with anomalous pathologies but no significant differences were found in relation to treatment with PM012. A mass observed in one female treated with 1,000 mg/kg/day was identified as a fibroadenoma, a benign tumor derived from mammary epithelium and connective tissue. It is a spontaneous tumor that is commonly observed in rats [37-39]. In the hematological and serum biochemistry tests, all changes observed in the female recovery group and main groups of both sexes were within normal levels and no significant changes were found in the related test parameters. In terms of organ weights, the increases of the absolute and relative weights of the thymus observed in the female 2,000 mg/kg/day recovery group were similar to those in the 4-week repeated dose toxicity study, and a dose-related increasing trend was observed in the female main groups. However, no other abnormal changes of the thymus were observed in the related test parameters of the histopathological examination, and therefore the changes were deemed not toxicologically significant [40]. In the necropsy findings, changes observed in the main and recovery groups were spontaneous or agonal congestion/hemorrhage due to anesthesia and not related to the administration of PM012.

In the histopathological examination, no noteworthy PM012-related lesions were observed, though some abnormalities were found. The lesions observed in the kidneys, such as glomenulonephropathy, basophilic tubules in the kidneys and hyaline cast, were high in frequency, primarily in males. However, these symptoms are part of chronic progressive nephropathy often observed in rats of approximately 5 months to old age and is particularly frequently observed in males. This nephropathy is accompanied by hyaline cast, which most frequently occurs due to an increase of glomerular penetrability or disorder renal tubular reabsorption. Multifocal alveolar histocytosis was observed in the lungs and is generally characterized by the infiltration of alveolar macrophages with foamy cytoplasm in the pulmonary alveoli near terminal bronchial tubes. This alveolar histocytosis is known to appear when drugs such as aminodarone and iprindole are administered, but it also is known to occur spontaneously in the lungs of rats. All lesions observed in the kidneys and lungs described above were not considered to be changes triggered by the administration of PM012 based on the comparison to the vehicle control group [38,39,41]. Furthermore, the diffuse cortical vacuolation observed in the zona fasciculate of the male adrenal gland, the accessory adrenocortical tissue observed in the female adrenal gland, the cyst and pseudocyst observed in the distal pituitary gland, the focal myocarditis observed only in the male heart, the follicular cyst and corpus luteum cyst observed in female ovaries are all spontaneous lesions [38,39,41]. All of these conditions are readily detected in old rats and were not considered to be changes triggered by the administration of PM012 based on the comparison to the vehicle control group.

Other anomalies were determined to be unrelated to the administration of PM012. In females, an enlargement of uterine cavity, hydrometra, was observed in similar frequency in the vehicle control group, and because no other lesions were observed in the uterus, such as in the endometrial epithelium or myometrial area, it was judged that these changes were not triggered by the administration of PM012 and were considered as changes related to the regular sexual cycle [41]. No cellular reaction to a hemorrhage in the parenchyma of the thymus was observed, and a hemorrhage is a lesion that is often seen after etherization and therefore was not considered to be an effect of the administration of PM012 [41]. A benign ganglioneuroma derived from adrenal medulla cells and consisting of histopathologically proliferative big ganglion cells and interstitial nervous tissue such as Schwann cells, satellite cells and nerve fibers was observed in a male treated with 2,000 mg/kg/day. The tumors observed in this study were observed to be over 80% ganglioneuromas and the rest had the histological features of pheochromocytomas. In this case, it is reasonable to diagnose these tumors as ganglioneuromas rather than complex pheochromocytomas, as this type of tumors is seldom found in old rats [38]. The fibroadenoma observed in the mammary gland of the skin in a female from the middle dose group was a benign tumor derived from the mammary epithelium and connective tissue, which is observed at a high frequency in rats and was judged to have occurred spontaneously and therefore was unrelated to treatment with PM012 [38,39]. We also found that the 4- or 26-week repeated oral administration of PM012 in male and female rats induced an increase and increasing trend in the weight of the thymus in the female treatment groups (main and recovery groups), but the change was judged to be toxicologically insignificant.

## Conclusion

Given our evaluation of all the data, the no observed adverse effects levels (NOAEL) of PM012 was determined to be 2,000 mg/kg/day for both sexes, and the target organ was not identified. We conclude that PM012 has potential for use in the treatment of the Alzheimer's disease without serious adverse effects.

## Abbreviations

hPS2m: Human presenilin 2 mutant transgenic mice; AD: Alzheimer's disease; ACh: Acetylcholine; ChAT: Choline acetyltransferase; *β-APP*: β-amyloid precursor protein; PS1: Presenilin 1; PS2: Presenilin 2; *APOE-E4*: Apolipoprotein E type 4; YMJ: Yukmijihwang-tang or Luweidihuang-wang; PM012: YMJ derivatives; WBC: White blood cell; RBC: Red blood cell; HGB: Hemoglobin concentration; HCT: Hematocrit; MCV: Mean corpuscular volume; MCH: Mean corpuscular hemoglobin; MCHC: Mean corpuscular hemoglobin concentration; RDW: Red cell distribution width; PLT: Platelet; MPV: Mean platelet volume; RET: Reticulocyte; NEU: Neutrophil; LYM: Lymphocyte; MONO: Monocyte; EOS: Eosinophil; BASO: Basophil; AST: Aspartate aminotransferase; ALT: Alanine aminotransferase; ALP: Alkaline phosphatase; CPK: Creatine phosphokinase; BIL: Total bilirubin; GLU: Glucose; CHO: Total cholesterol; TG: Triglyceride; PRO: Total protein; ALB: Albumin; A/G ratio: Albumin/Globulin ratio; BUN: Blood urea nitrogen; CRE: Creatinine; IP: Inorganic phosphorus.

## Competing interests

The authors declare that they have no competing interests.

## Authors' contributions

All authors participated in the acquisition of data and revision of the manuscript. All authors conceived of the study, determined the design, interpreted the data and drafted the manuscript. All authors read and gave final approval for the version submitted for publication.

## Pre-publication history

The pre-publication history for this paper can be accessed here:

http://www.biomedcentral.com/1472-6882/12/24/prepub

## Supplementary Material

Additional file 1 **Table S1**. Urinalysis in rats orally treated with PM012. Click here for file
