# Supplementary material for: Safety and efficacy assessment of standardized herbal formula PM012
Source: BMC Complement Altern Med. 2012 Mar 29;12:24. doi: 10.1186/1472-6882-12-24 (PMC3342231; doi:10.1186/1472-6882-12-24)
Supplement: Additional file 1 — Table S1. Urinalysis in rats orally treated with PM012. [file 1472-6882-12-24-S1.DOC]

Table 1. Urinalysis in rats orally treated with PM012

| Parameter | Results | Grade | Dose (mg/kg) | | | | | | | | | | | | | | | | | | | | | | |
| --- | --- | --- | --- | --- | --- | --- | --- | --- | --- | --- | --- | --- | --- | --- | --- | --- | --- | --- | --- | --- | --- | --- | --- | --- | --- |
|  |  |  | 0 | | | | | | 500 | | | | | 1000 | | | | | 2000 | | | | | | |
|  |  |  | 4 Weeks | | 26 Weeks | | Recovery | | | 4 Weeks | | 26 Weeks | | | 4 Weeks | | 26 Weeks | | | 4 Weeks | | 26 Weeks | | Recovery | |
|  |  |  | M | F | M | F | M | F | | M | F | M | F | | M | F | M | F | | M | F | M | F | M | F |
| GLU  BIL  KET  SG  pH  PRO  URO  NIT  OB  WBC | -  ±  -  -  ±  1+  ≤1.005  1.010  1.015  1.020  1.025  ≥1.030  6.0  6.5  7.0  7.5  8.0  8.5  -  ±  1+  2+  3+  0.1  -  -  ±  1+  2+  3+  -  ±  1+  2+  3+ | 0  1  0  0  1  2  0  1  2  3  4  5  0  1  2  3  4  5  0  1  2  3  4  0  0  0  1  2  3  4  0  1  2  3  4 | 5  0  5  3  0  2  0  0  0  0  0  5  0  0  2  2  1  0  1  1  1  0  2  5  5  4  0  1  0  0  1  2  0  2  0 | 5  0  5  5  0  0  0  0  1  1  0  3  1  0  0  2  2  0  3  1  1  0  0  5  5  5  0  0  0  0  3  2  0  0  0 | 5  0  5  5  0  0  2  0  2  1  0  0  0  0  0  1  4  0  0  0  0  2  3  5  5  2  3  0  0  0  0  0  0  4  1 | 5  0  5  5  0  0  0  4  1  0  0  0  0  0  0  3  2  0  3  1  0  1  0  5  5  5  0  0  0  0  2  0  2  1  0 | 5  0  5  1  4  0  0  0  0  0  4  1  0  0  1  1  2  1  0  0  0  0  5  5  5  3  2  0  0  0  0  0  0  5  0 | 5  0  5  5  0  0  0  0  2  2  1  0  0  0  1  2  2  0  1  2  1  1  0  5  5  3  1  1  0  0  1  2  2  0  0 | | 5  0  5  4  1  0  0  0  0  0  0  5  1  0  3  1  0  0  0  0  0  1  4  5  5  4  0  1  0  0  4  0  1  0  0 | 5  0  5  5  0  0  0  0  1  0  2  2  0  0  0  2  3  0  3  1  0  1  0  5  5  5  0  0  0  0  4  1  0  0  0 | 5  0  5  5  0  0  1  3  0  1  0  0  0  0  0  2  3  0  0  2  1  1  1  5  5  4  1  0  0  0  1  0  2  1  1 | 5  0  5  5  0  0  0  1  3  1  0  0  0  0  1  2  2  0  2  1  1  0  1  5  5  4  0  0  0  1  2  0  2  1  0 | | 5  0  5  2  3  0  0  0  0  0  0  5  1  1  3  0  0  0  0  0  1  2  2  5  5  4  0  1  0  0  1  1  2  1  0 | 5  0  5  4  1  0  0  0  0  2  2  1  1  0  3  1  0  0  1  0  3  1  0  5  5  5  0  0  0  0  4  1  0  0  0 | 5  0  5  5  0  0  1  0  2  2  0  0  0  0  0  1  4  0  0  0  2  1  2  5  5  4  1  0  0  0  0  1  1  2  1 | 5  0  5  5  0  0  0  1  1  3  0  0  0  0  0  1  4  0  1  1  2  1  0  5  5  4  1  0  0  0  0  2  0  2  1 | | 5  0  5  2  1  2  0  0  0  0  0  5  1  1  1  2  0  0  0  0  1  0  4  5  5  4  0  1  0  0  2  0  1  2  0 | 5  0  5  4  1  0  0  0  1  0  0  4  3  0  1  1  0  0  1  2  1  1  0  5  5  5  0  0  0  0  3  0  1  1  0 | 5  0  5  5  0  0  0  1  3  0  1  0  0  0  0  0  5  0  0  0  0  4  1  5  5  3  1  1  0  0  0  0  2  3  0 | 4  1  5  5  0  0  1  1  1  2  0  0  0  0  0  3  2  0  2  2  0  1  0  5  5  4  0  0  0  1  2  2  0  1  0 | 5  0  5  1  4  0  0  0  0  2  3  0  0  0  1  1  2  1  0  0  0  0  5  5  5  3  1  1  0  0  0  0  0  3  2 | 5  0  5  5  0  0  0  0  3  1  1  0  0  0  0  1  4  0  3  1  0  1  0  5  5  5  0  0  0  0  3  2  0  0  0 |
| N | | | 5 | 5 | 5 | 5 | 5 | 5 | | 5 | 5 | 5 | 5 | | 5 | 5 | 5 | 5 | | 5 | 5 | 5 | 5 | 5 | 5 |

GLU:Glucose, BIL: Bilirubin, KET: Ketone body, SG: Specific gravity, PRO: Protein, URO: Urobilinogen, NIT: Nitrite, OB: Occult blood, WBC: Leukocyte, M:male, F: female

Values are mean ±SD (n=5 (4weeks or 26weks or recovery)/sex/dose).
